# Cooking fuels and risk of all-cause and cardiopulmonary mortality in urban China: a prospective cohort study

**DOI:** 10.1016/S2214-109X(19)30525-X

**Published:** 2020-01-20

**Authors:** Kuai Yu, Jun Lv, Gaokun Qiu, Canqing Yu, Yu Guo, Zheng Bian, Ling Yang, Yiping Chen, Chaolong Wang, An Pan, Liming Liang, Frank B Hu, Zhengming Chen, Liming Li, Tangchun Wu, China Kadoorie Biobank Study, Junshi Chen, Zhengming Chen, Robert Clarke, Rory Collins, Yu Guo, Liming Li, Jun Lv, Richard Peto, Robin Walters, Daniel Avery, Ruth Boxall, Yumei Chang, Yiping Chen, Zhengming Chen, Robert Clarke, Huaidong Du, Simon Gilbert, Alex Hacker, Mike Hill, Michael Holmes, Andri Iona, Christiana Kartsonaki, Rene Kerosi, Ling Kong, Om Kurmi, Garry Lancaster, Sarah Lewington, Kuang Lin, John McDonnell, Iona Millwood, Qunhua Nie, Jayakrishnan Radhakrishnan, Paul Ryder, Sam Sansome, Dan Schmidt, Paul Sherliker, Rajani Sohoni, Becky Stevens, Iain Turnbull, Robin Walters, Jenny Wang, Lin Wang, Neil Wright, Ling Yang, Xiaoming Yang, Zheng Bian, Yu Guo, Xiao Han, Can Hou, Jun Lv, Pei Pei, Chao Liu, Yunlong Tan, Canqing Yu, Zengchang Pang, Ruqin Gao, Shanpeng Li, Shaojie Wang, Yongmei Liu, Ranran Du, Yajing Zang, Liang Cheng, Xiaocao Tian, Hua Zhang, Yaoming Zhai, Feng Ning, Xiaohui Sun, Feifei Li, Silu Lv, Junzheng Wang, Wei Hou, Mingyuan Zeng, Ge Jiang, Xue Zhou, Liqiu Yang, Hui He, Bo Yu, Yanjie Li, Qinai Xu, Quan Kang, Ziyan Guo, Dan Wang, Ximin Hu, Hongmei Wang, Jinyan Chen, Yan Fu, Zhenwang Fu, Xiaohuan Wang, Min Weng, Zhendong Guo, Shukuan Wu, Yilei Li, Huimei Li, Zhifang Fu, Ming Wu, Yonglin Zhou, Jinyi Zhou, Ran Tao, Jie Yang, Jian Su, Fang Liu, Jun Zhang, Yihe Hu, Yan Lu, Liangcai Ma, Aiyu Tang, Shuo Zhang, Jianrong Jin, Jingchao Liu, Zhenzhu Tang, Naying Chen, Ying Huang, Mingqiang Li, Jinhuai Meng, Rong Pan, Qilian Jiang, Jian Lan, Yun Liu, Liuping Wei, Liyuan Zhou, Ningyu Chen, Ping Wang, Fanwen Meng, Yulu Qin, Sisi Wang, Xianping Wu, Ningmei Zhang, Xiaofang Chen, Weiwei Zhou, Guojin Luo, Jianguo Li, Xiaofang Chen, Xunfu Zhong, Jiaqiu Liu, Qiang Sun, Pengfei Ge, Xiaolan Ren, Caixia Dong, Hui Zhang, Enke Mao, Xiaoping Wang, Tao Wang, Xi Zhang, Ding Zhang, Gang Zhou, Shixian Feng, Liang Chang, Lei Fan, Yulian Gao, Tianyou He, Huarong Sun, Pan He, Chen Hu, Xukui Zhang, Huifang Wu, Pan He, Min Yu, Ruying Hu, Hao Wang, Yijian Qian, Chunmei Wang, Kaixu Xie, Lingli Chen, Yidan Zhang, Dongxia Pan, Qijun Gu, Yuelong Huang, Biyun Chen, Li Yin, Huilin Liu, Zhongxi Fu, Qiaohua Xu, Xin Xu, Hao Zhang, Huajun Long, Xianzhi Li, Libo Zhang, Zhe Qiu

**Affiliations:** a Department of Occupational and Environmental Health and Department of Epidemiology and Biostatistics, Key Laboratory of Environment and Health, Ministry of Education and State Key Laboratory of Environmental Health (Incubating), School of Public Health, Tongji Medical College, Huazhong University of Science and Technology, Wuhan, China; b Department of Epidemiology and Biostatistics, School of Public Health, Peking University Health Science Center, Beijing, China; c Chinese Academy of Medical Sciences, Beijing, China; d Clinical Trial Service Unit & Epidemiological Studies Unit (CTSU), Nuffield Department of Population Health, University of Oxford, Oxford, UK; e Department of Epidemiology and Department of Biostatistics, Harvard T H Chan School of Public Health, Boston, MA, USA; f Department of Nutrition and Department of Epidemiology, Harvard T H Chan School of Public Health, Boston, MA, USA

## Abstract

**Background:**

Cooking practice has transitioned from use of solid fuels to use of clean fuels, with addition of better ventilation facilities. However, the change in mortality risk associated with such a transition remains unclear.

**Methods:**

The China Kadoorie Biobank (CKB) Study enrolled participants (aged 30–79 years) from ten areas across China; we chose to study participants from five urban areas where transition from use of solid fuels to clean fuels for cooking was prevalent. Participants who reported regular cooking (weekly or more frequently) at baseline were categorised as persistent clean fuel users, previous solid fuel users, or persistent solid fuel users, according to self-reported fuel use histories. All-cause and cardiopulmonary mortality were identified through linkage to China's Disease Surveillance Point system and local mortality records.

**Findings:**

Between June 24, 2004, and July 15, 2008, 226 186 participants living in five urban areas of China were enrolled in the CKB Study. Among 171 677 participants who reported cooking regularly (weekly or more frequently), 75 785 (44%) were persistent clean fuel users, 80 511 (47%) were previous solid fuel users, and 15 381 (9%) were persistent solid fuel users. During a mean of 9·8 (SD 1·7) years of follow-up, 10 831 deaths were documented, including 3819 cardiovascular deaths and 761 respiratory deaths. Compared with persistent clean fuel users, persistent solid fuel users had significantly higher risks of all-cause mortality (hazard ratio [HR] 1·19, 95% CI 1·10–1·28), cardiovascular mortality (1·24, 1·10–1·39), and respiratory mortality (1·43, 1·10–1·85). The excess risk of all-cause and cardiopulmonary mortality fell by more than 60% in 5 years after cessation of solid fuel use and continued to decrease afterwards. Use of ventilation was associated with lower all-cause mortality risk, even among persistent clean fuel users (HR 0·78, 0·69–0·89).

**Interpretation:**

Solid fuel use for cooking is associated with a higher risk of mortality, and cessation of solid fuel use cuts excess mortality risks swiftly and substantially within 5 years. Ventilation use also lowers the risk of mortality, even among people who persistently use clean fuels. It is of prime importance for both policy makers and the public to accelerate the transition from solid fuels to clean fuels and promote efficient ventilation to minimise further adverse health effects.

**Funding:**

National Natural Science Foundation of China, Wellcome Trust, and Kadoorie Charitable Foundation.

## Introduction

Over the past few decades, rapid worldwide economic development has made different types of cooking fuel, including clean fuels such as gas and electricity, broadly accessible and affordable to the general population. Clean fuels, although higher in price, produce much less pollution than do traditional solid fuels, such as coal and biomass. Furthermore, various ventilation facilities have been designed to reduce pollution during cooking.^1^, ^2^, ^3^ Cooking with solid fuels is a major impediment to improvement of living standards for more than 2·7 billion people globally (450 million in China alone).^4^ Use of solid fuels for cooking accounts for approximately 1·6 million deaths worldwide, as estimated in the Global Burden of Diseases, Injuries, and Risk Factors Study (GBD) 2018,^5^, ^6^ and could lead to more deaths because cooking with solid fuels also contributes more than 12% to ambient air pollution.^7^, ^8^ The mortality burden could be further underestimated because of the integrated exposure-response approach used in GBD in areas with a high prevalence of solid fuel use, such as China.^9^ Indeed, in a study done in rural China, solid fuel use was associated with increased risk of all-cause and cardiovascular mortality and resulted in a much higher mortality burden than was estimated in GBD.^10^ Urban China, which is more economically developed than rural China and has seen a large-scale transition from use of solid fuels to clean fuels since the 1980s,^11^, ^12^, ^13^, ^14^ provides an unprecedented scenario to examine how risks for mortality have changed over time with the transition in fuel use and increase in use of ventilation.

Findings of several prospective studies^10^, ^15^, ^16^ have reported lower risks of all-cause, cardiovascular, and respiratory mortality after cessation of solid fuel use, but the relative risks associated with years since cessation remain unclear. To our knowledge, only one study of 74 941 women in Shanghai^16^ has reported the association between time since cessation of solid fuel use (coal use specifically) and mortality risks. In that study, the risk of ischaemic heart disease mortality was reduced to the level of never-users of coal after more than 20 years of cessation. However, risks for all-cause mortality or deaths from other causes were not associated with cessation of coal use compared with never-use of coal, even among people within 10 years after cessation,^16^ indicating that the risk reduction could have occurred soon after cessation and needed to be examined at finer timescales. If a substantial risk reduction was proven to occur within only a few years after cessation of solid fuel use, implementation of clean fuel initiatives might be prioritised and better motivated. Moreover, the Shanghai study was restricted to women living in the most economically developed city in China,^16^ and only about 1% of participants were persistent coal users; therefore, the relative mortality risk for cessation of coal use compared with persistent coal use remains unclear. The effects on health of ventilation during cooking with fuel types other than solid fuels are also important to investigate.^17^ Mortality risks are diminished when ventilation is used,^10^, ^15^ since even clean fuel is not entirely exempt from producing pollutants, such as fine particulate matter (PM_2·5_).^18^

**Research in context**

**Evidence before this study**

We searched PubMed, Web of Science, and Google Scholar up to Oct 24, 2019, with the keywords: (“cooking fuel” OR “solid fuel” OR “coal” OR “wood”) AND (“cessation” OR “switch” OR “transition”) AND (“mortality” OR “total death” OR “all-cause death” OR “cardiovascular mortality” OR “respiratory mortality”). We searched for articles and reviews with search terms in English, but we did not apply any language restrictions. Our search retrieved three prospective cohort studies that have assessed the association between cessation of solid fuel use and mortality risk. Two studies provided preliminary evidence that mortality risk was lower after cessation of solid fuel use, without examining the time course of risk reduction. In the third study of 74 941 women from Shanghai, mortality risk was investigated in association with cessation of coal use for fewer than 10 years, 10–20 years, and more than 20 years; a higher risk of ischaemic heart disease mortality was associated with cessation of coal use, with the risk falling with increasing years after cessation. However, no increased risk was noted for all-cause mortality and death from other causes, even among the group within 10 years of cessation, indicating that risk reduction could have occurred quickly after cessation and needed to be examined at finer time scales. If a substantial risk reduction was proven to occur within only a few years after cessation of solid fuel use, implementation of clean fuel initiatives would be prioritised and better motivated. Moreover, only about 1% of the participants in the Shanghai study were persistent coal fuel users, leading to an inaccurate estimate of the relative risk of cessation of coal use compared with persistent coal use. Moreover, none of the three studies investigated the associations separately among men and women, or the effect of ventilation on mortality risks among participants cooking with clean fuels.

**Added value of this study**

Our study, with nearly 10 years of follow-up, included 171 677 participants from five urban areas across China who reported that they cooked regularly. Our findings showed for the first time that the excess risks of all-cause and cardiopulmonary mortality from use of solid cooking fuels decreased by more than 60% at 5 years after cessation, and continued to decrease afterwards. Our study extends previous findings to men and women separately, and confirms similar results in both sexes on the relations between cessation of solid fuel use and mortality risks. Moreover, our study provides novel evidence that ventilation use is associated with lower mortality risks, even among people who use clean cooking fuels. To our knowledge, our study is the largest and most comprehensive study to date investigating the association of cooking fuel types, time since switching to clean fuels, and use of ventilation with risks of all-cause and cardiopulmonary mortality.

**Implications of all the available evidence**

Our findings underscore the importance of improving access to both clean fuels and ventilation to reduce global mortality burden from cooking fuel use. The greatest reductions in risks of mortality occurred within the first 5 years after cessation. 2·7 billion people are still using solid fuels for cooking globally; therefore, the transition from solid fuels to clean fuels, and use of ventilation, are likely to yield substantial environmental improvements and public health gains.

Using prospective data from the China Kadoorie Biobank (CKB) Study, we aimed to investigate associations between types of cooking fuel, time since switching to clean fuels, use of ventilation, and risks of all-cause and cardiopulmonary mortality.

## Methods

### Study design and population

Details of the CKB Study have been described elsewhere.^19^, ^20^ Briefly, the CKB Study recruited participants (aged 30–79 years) from ten geographically diverse areas across China. Every participant answered a questionnaire administered by a trained interviewer, to gather information on sociodemographic characteristics, lifestyle factors, household air pollution exposures, and medical history. After completion of the baseline survey, a small proportion of participants was randomly chosen from the ten study areas and resurveyed to check the reproducibility of baseline information.

Because rural and urban populations in China have strikingly different energy use patterns and are at distinct phases of fuel use transition,^12^ we chose to focus our study on CKB Study participants from five urban areas (appendix p 8) where the transition from use of solid fuels to clean fuels for cooking was prevalent.

The CKB study was approved by the Oxford University Tropical Research Ethics Committee and the Chinese Center for Disease Control and Prevention Ethics Review Committee.^19^ All participants provided written informed consent.

### Procedures

We gathered information from participants on cooking frequency, the primary fuel used for cooking, cookstove ventilation, and years lived in each of the three most recent residences. Among the primary cooking fuels reported, coal and wood were defined as solid fuels whereas gas and electricity were defined as clean fuels. Participants who reported cooking daily or weekly were further categorised according to their history of cooking fuel use before baseline, as either persistent users of clean fuel, previous users of solid fuel (participants who reported using clean fuel at baseline but used solid fuels in one or more previous residence), or persistent users of solid fuel. Among previous users of solid fuel, the time since cessation of solid fuel use was calculated by aggregating the time (in years) lived in consecutive residences during which time clean fuel was the primary cooking fuel, assuming that the primary fuel used for cooking had not changed during each residential period. We defined the people who cooked with ventilation as those whose cookstove in the present residence was equipped with a chimney or a kitchen exhaust fan.

We gathered information on all-cause and cardiopulmonary mortality periodically (from baseline until Dec 31, 2016) through linkage to China's Disease Surveillance Point system via each participant's unique identification number, supplemented by the national health insurance system and annual active confirmation of survival obtained by local street committees and village administrators.^21^ The underlying cause of death was classified by trained staff who were unaware of baseline information, using the International Statistical Classification of Diseases and Related Health Problems, 10th Revision (ICD-10).^22^

The primary outcomes of our study were all-cause mortality (ICD-10 codes 001–999), cardiovascular disease mortality (I00–I25, I27–I88, and I95–I99), and respiratory disease mortality (J00–J99). Secondary endpoints were the main components of cardiopulmonary mortality, including ischaemic heart disease (I20–I25), stroke (I60–I61 and I63–I64), ischaemic stroke (I63), haemorrhagic stroke (I61), chronic obstructive pulmonary disease (COPD; J41–J44), and pneumonia (J12–J18).

### Statistical analysis

Baseline characteristics of the study population are presented as means with SDs or percentages, by type of cooking fuel use. The reproducibility of baseline information, including cooking fuel use, was assessed by intraclass correlation coefficients (ICC) for continuous variables and the weighted κ statistic^23^ for categorical variables, using repeated measures at baseline and in the resurvey (mean 2·5 [SD 0·6] years after baseline). Cox proportional-hazards regression models were used to estimate multivariate-adjusted hazard ratios (HRs) and 95% CIs of mortality risks associated with type of cooking fuel use, time since cessation of solid fuel use, and use of ventilation. We tested the proportional-hazard assumption using Schoenfeld residuals^24^ and found no evidence of departure from the assumption in models for all-cause mortality (p=0·079), cardiovascular mortality (p=0·081), and respiratory mortality (p=0·26).

We used the restricted cubic spline function in a stratified Cox model to inspect the change of mortality risk along with time since cessation of use of solid fuel, with the %LGTPHCURV9 macro in SAS version 9.4.^25^ We set three knots at the 5th, 50th, and 95th percentiles of the exposure variable (ie, time since cessation of solid fuel use) for the restricted cubic spline function, with no offset values for the fixed terms, resulting in two degrees of freedom (2 df). Non-linearity was examined by a likelihood ratio test, which compared two models with and without non-linear terms. We also did separate analyses among men and women and across subgroups of smoking status, considering the possible joint effect of smoking and solid fuel use on mortality risk.^10^ In all analyses, exposure variables were assessed using information on fuel use reported at baseline without considering any changes in fuel use during follow-up.

To test the robustness of our HR estimates of mortality risks associated with types of cooking fuel, we did five sensitivity analyses. First, we made further adjustments for conventional cardiovascular risk factors or other potential confounders.^26^, ^27^ Second, we excluded participants with major diseases (including cardiovascular disease and cancer) at baseline. Third, we excluded participants who died within the first 2 years of follow-up, to scrutinise reverse causality; this analysis would look at the possibility that these participants had baseline subclinical diseases that could affect fuel choice. Fourth, we analysed coal use and wood use separately. Finally, we looked at specific causes of death, including ischaemic heart disease, stroke (and ischaemic and haemorrhagic subtypes of stroke), COPD, and pneumonia.

We judged two-sided p values less than 0·05 statistically significant. All analyses were done using SAS version 9.4. Graphs were plotted using R version 3.4.2. Model adjustments and other details about statistical analyses are provided in the appendix (p 3).

### Role of the funding source

The funders had no role in study design, data collection, data analysis, data interpretation, or writing of the report. The corresponding authors had full access to all data in the study and had final responsibility for the decision to submit for publication.

## Results

Between June 25, 2004, and July 15, 2008, 512 891 adults from ten areas of China (five rural and five urban) completed the baseline questionnaire for the CKB Study and provided physical measurements. Between May 26, 2008, and Oct 10, 2008, 19 788 (4%) participants were randomly chosen for resurveying, to check the reproducibility of baseline information. By Dec 31, 2016, 4781 (1%) of 512 891 participants recruited to the CKB Study at baseline had been lost to follow-up, and 44 037 (9%) had died.

226 186 participants were enrolled from five urban areas of China. 179 participants were excluded from our study because the total duration of their three most recent residential periods was greater than their age; 54 317 participants were excluded because they did not cook regularly (monthly [n=9126]; rarely or never [n=45 191]), and 74 participants were excluded because they used other unspecified fuels for cooking (appendix p 9).

171 677 (76%) participants reported cooking regularly (weekly or more frequently) at baseline, of whom 75 785 (44%) were persistent users of clean fuel, 80 511 (47%) were previous users of solid fuel, and 15 381 (9%) were persistent users of solid fuel (table 1). Among both men and women, previous and persistent solid fuel users were older and less educated, had lower household income and worse self-reported health status, and were less likely to use cookstove ventilation (table 1). Data for 8161 (4%) participants included in the resurvey are presented in the appendix (p 4). Baseline information, including education level, household income, smoking, drinking, heating fuel use, ventilation status, physical activity and body-mass index, showed reasonable agreement with that reported or measured in the resurvey (ICC ≥0·74 for continuous variables; weighted κ values ≥0·43 for categorical variables). In particular, 6431 (79%) and 6594 (81%) participants in the resurvey reported the same cooking fuel use and ventilation use as at baseline, yielding weighted κ values of 0·56 and 0·55, respectively (appendix p 4).

**Table 1:** Baseline characteristics of study participants according to types of cooking fuel

		**Overall (n=171 677)**			**Women (n=121 366)**			**Men (n=50 311)**		
		Persistent clean fuel users (n=75 785)	Previous solid fuel users (n=80 511)	Persistent solid fuel users (n=15 381)	Persistent clean fuel users (n=47 077)	Previous solid fuel users (n=62 957)	Persistent solid fuel users (n=11 332)	Persistent clean fuel users (n=28 708)	Previous solid fuel users (n=17 554)	Persistent solid fuel users (n=4049)
Age (years)		49·2 (10·1)	55·6 (9·9)	57·1 (10·6)	48·4 (9·6)	55·6 (9·9)	56·7 (10·8)	50·4 (10·8)	55·7 (9·8)	58·2 (10·2)
Education										
	Primary school or lower	12 013 (16%)	34 791 (43%)	11 469 (75%)	8333 (18%)	29 234 (46%)	8991 (79%)	3680 (13%)	5557 (32%)	2478 (61%)
	Middle school	25 725 (34%)	26 160 (32%)	2933 (19%)	15 296 (32%)	19 521 (31%)	1774 (16%)	10 429 (36%)	6639 (38%)	1159 (29%)
	High school or higher	38 047 (50%)	19 560 (24%)	979 (6%)	23 448 (50%)	14 202 (23%)	567 (5%)	14 599 (51%)	5358 (31%)	412 (10%)
Household income per year (¥)*										
	<20 000	28 582 (38%)	37 624 (47%)	10 573 (69%)	17 984 (38%)	30 492 (48%)	8059 (71%)	10 598 (37%)	7132 (41%)	2514 (62%)
	20 000–34 999	26 791 (35%)	24 934 (31%)	3037 (20%)	16 470 (35%)	19 120 (30%)	2141 (19%)	10 321 (36%)	5814 (33%)	896 (22%)
	≥35 000	20 412 (27%)	17 953 (22%)	1771 (12%)	12 623 (27%)	13 345 (21%)	1132 (10%)	7789 (27%)	4608 (26%)	639 (16%)
Smoking category										
	Never-smoker	50 966 (67%)	62 321 (77%)	11 623 (76%)	45 430 (97%)	59 614 (95%)	11 061 (98%)	5536 (19%)	2707 (15%)	562 (14%)
	Former smoker	4214 (6%)	3818 (5%)	709 (5%)	182 (<1%)	720 (1%)	41 (<1%)	4032 (14%)	3098 (18%)	668 (16%)
	Current smoker	20 605 (27%)	14 372 (18%)	3049 (20%)	1465 (3%)	2623 (4%)	230 (2%)	19 140 (67%)	11 749 (67%)	2819 (70%)
Heating fuel use										
	No heating	26 642 (35%)	41 741 (52%)	13 214 (86%)	17 090 (36%)	31 594 (50%)	9794 (86%)	9552 (33%)	10 147 (58%)	3420 (84%)
	Clean fuel	35 901 (47%)	30 549 (38%)	721 (5%)	21 711 (46%)	24 446 (39%)	485 (4%)	14 190 (49%)	6103 (35%)	236 (6%)
	Solid fuel	13 201 (17%)	8184 (10%)	1445 (9%)	8252 (18%)	6884 (11%)	1052 (9%)	4949 (17%)	1300 (7%)	393 (10%)
	Unspecified fuel	41 (<1%)	37 (<1%)	1 (<1%)	24 (<1%)	33 (<1%)	1 (<1%)	17 (<1%)	4 (<1%)	0 (0%)
Ever-drinker		48 213 (64%)	37 140 (46%)	4257 (28%)	23 061 (49%)	22 118 (35%)	1212 (11%)	25 152 (88%)	15 022 (86%)	3045 (75%)
Physical activity (MET-h per day)†		19·0 (11·5)	17·9 (12·4)	20·7 (13·9)	18·2 (10·9)	17·1 (11·6)	19·7 (12·5)	20·2 (12·4)	20·9 (14·6)	23·5 (17·0)
Body-mass index (kg/m^2^)		24·4 (3·4)	24·6 (3·4)	22·9 (3·4)	24·2 (3·4)	24·6 (3·5)	23·0 (3·5)	24·7 (3·3)	24·3 (3·2)	22·8 (3·1)
Waist circumference (cm)		81·9 (9·9)	81·9 (9·6)	77·4 (9·5)	79·2 (9·2)	81·2 (9·5)	77·0 (9·4)	86·2 (9·4)	84·3 (9·6)	78·6 (9·6)
Systolic blood pressure (mm Hg)		126·3 (20·3)	131·3 (21·4)	132·0 (23·1)	122·9 (20·1)	130·5 (21·8)	131·3 (23·7)	131·8 (19·3)	134·1 (19·8)	133·9 (21·1)
Hypertension		21 386 (28%)	31 267 (39%)	5628 (37%)	11 168 (24%)	23 847 (38%)	4004 (35%)	10 218 (36%)	7420 (42%)	1624 (40%)
Diabetes		5232 (7%)	7712 (10%)	772 (5%)	3014 (6%)	6151 (10%)	572 (5%)	2218 (8%)	1561 (9%)	200 (5%)
Poor self-reported health‡		5052 (7%)	8578 (11%)	1957 (13%)	3230 (7%)	7068 (11%)	1502 (13%)	1822 (6%)	1510 (9%)	455 (11%)
Passive smoking		42 234 (56%)	43 628 (54%)	8965 (58%)	25 115 (53%)	33 147 (53%)	6414 (57%)	17 119 (60%)	10 481 (60%)	2551 (63%)
Cookstove ventilation		70 411 (93%)	71 887 (89%)	10 592 (69%)	43 753 (93%)	56 303 (89%)	7538 (67%)	26 658 (93%)	15 584 (89%)	3054 (75%)

Data (continuous variables) are mean (SD) or (categorical variables) percentages.

* At the exchange rate as of November, 2019, ¥100 is approximately equal to US$£15.

† MET-h per day=metabolic equivalent of task h per day. 1 MET-h is defined as 1 kcal/kg per h.

‡ Participants were asked to rate their current general health status, with choices of excellent, good, fair, and poor.

Mean follow-up, defined as the time between the baseline examination and death or the censor date (Dec 31, 2016), was 9·8 (SD 1·7) years for the 171 677 participants analysed. During follow-up, 10 831 deaths were recorded, including 3819 cardiovascular deaths and 761 respiratory deaths. Compared with persistent users of clean fuel, persistent users of solid fuel had an increased risk of all-cause mortality (HR 1·19, 95% CI 1·10–1·28), cardiovascular mortality (1·24, 1·10–1·39), and respiratory mortality (1·43, 1·10–1·85). For previous users of solid fuel, the risk of all-cause mortality compared with that of persistent clean fuel users was increased among those who had ceased use of solid fuel within the past 5 years (HR 1·07, 95% CI 1·01–1·14), but risk did not show statistical significance among those with cessation of use for 5–10 years (1·03, 0·93–1·13) or for more than 10 years (0·94, 0·89–1·01; table 2). Similar associations were recorded when analyses were stratified by sex (table 2) or study area (appendix p 10) and in sensitivity analyses adjusting for additional potential risk factors or excluding participants with major diseases or who died within the first 2 years of follow-up (appendix p 5). In particular, analyses excluding participants who died within the first 2 years of follow-up yielded almost the same results as in the main analyses, indicating that our results were statistically robust. To gain additional insights, further analyses were done of exposure to subtypes of solid fuels and mortality caused by different cardiopulmonary diseases. Users of wood for cooking generally had higher risks of all-cause and cardiopulmonary mortality than did coal users, except for risk of respiratory mortality in women (appendix p 6). Furthermore, persistent users of solid fuel were at increased risk of mortality from all stroke, ischaemic stroke, haemorrhagic stroke, and COPD, but not for ischaemic heart disease and pneumonia (appendix p 7).

**Table 2:** Adjusted hazard ratios for all-cause and cardiopulmonary mortality by sex and type of cooking fuel

		**N**	**All-cause mortality**		**Cardiovascular mortality**		**Respiratory mortality**	
			Deaths (n)	Hazard ratio (95% CI)	Deaths (n)	Hazard ratio (95% CI)	Deaths (n)	Hazard ratio (95% CI)
**Total**								
Persistent clean fuel users		75 785	3608	1 (ref)	1235	1 (ref)	209	1 (ref)
Previous solid fuel users		80 511	5752	0·97 (0·93–1·01)	2081	0·98 (0·91–1·05)	415	1·08 (0·90–1·29)
	<5 years since cessation	7728	579	1·07 (1·01–1·14)	193	1·12 (0·92–1·39)	40	1·19 (0·91–1·57)
	5-10 years since cessation	17 927	1184	1·03 (0·93–1·13)	392	0·97 (0·86–1·09)	80	1·11 (0·85–1·45)
	>10 years since cessation	54 856	3989	0·94 (0·89–1·01)	1496	0·98 (0·90–1·06)	295	1·09 (0·90–1·32)
Persistent solid fuel users		15 381	1471	1·19 (1·10–1·28)	503	1·24 (1·10–1·39)	137	1·43 (1·10–1·85)
**Women**								
Persistent clean fuel users		47 077	1335	1 (ref)	410	1 (ref)	66	1 (ref)
Previous solid fuel users		62 957	3910	1·04 (0·97–1·11)	1469	1·10 (0·98–1·23)	250	0·98 (0·74–1·30)
	<5 years since cessation	5867	362	1·10 (1·01–1·21)	132	1·13 (0·92–1·40)	21	1·17 (0·64–2·13)
	5-10 years since cessation	13 803	785	1·05 (0·93–1·18)	267	1·08 (0·92–1·27)	41	0·87 (0·58–1·30)
	>10 years since cessation	43 287	2763	1·01 (0·94–1·08)	1070	1·02 (0·88–1·22)	188	0·98 (0·73–1·32)
Persistent solid fuel users		11 332	876	1·25 (1·14–1·38)	322	1·38 (1·17–1·63)	77	1·40 (0·96–2·04)
**Men**								
Persistent clean fuel users		28 708	2273	1 (ref)	825	1 (ref)	143	1 (ref)
Previous solid fuel users		17 554	1842	0·94 (0·88–1·00)	612	0·91 (0·82–1·02)	165	1·18 (0·94–1·49)
	<5 years since cessation	1861	217	1·03 (0·89–1·19)	61	1·08 (0·83–1·43)	19	1·25 (0·63–2·54)
	5-10 years since cessation	4124	399	0·97 (0·87–1·08)	125	0·88 (0·73–1·07)	39	1·36 (0·83–2·23)
	>10 years since cessation	11 569	1226	0·93 (0·86–1·00)	426	0·90 (0·80–1·02)	107	1·12 (0·87–1·46)
Persistent solid fuel users		4049	595	1·11 (1·00–1·22)	181	1·16 (0·97–1·38)	60	1·48 (1·06–2·05)

Hazard ratios were derived from Cox models stratified by age at risk, sex (when appropriate), and study area and adjusted for education level, household income, alcohol consumption, smoking status, passive smoking, physical activity, body-mass index, diet (consumption of fresh fruit, preserved vegetables, and meat), cookstove ventilation, and solid fuel use for heating.

**Figure 1:**
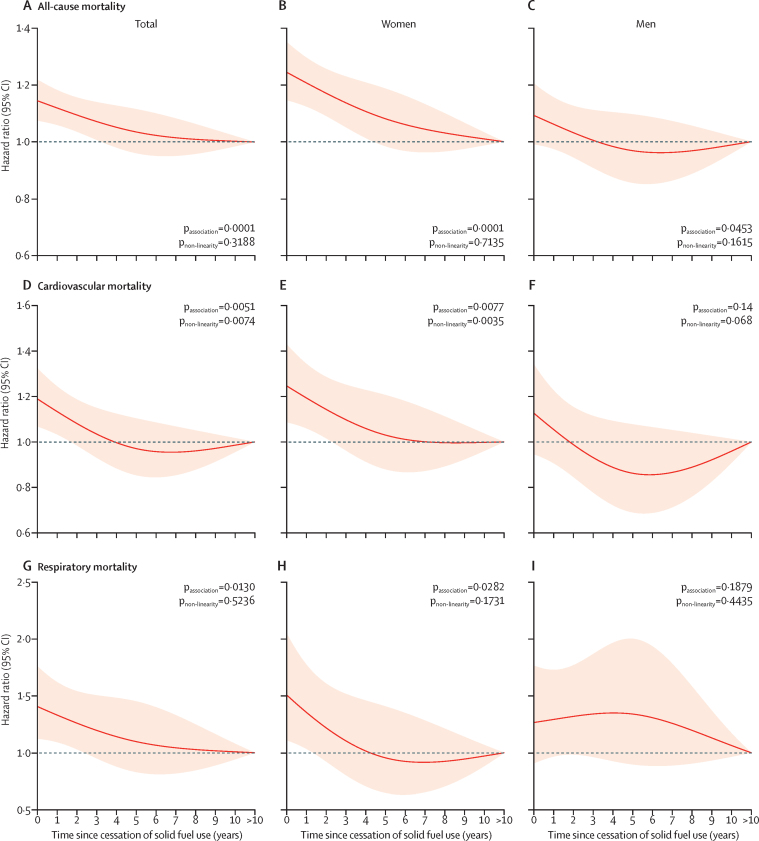
Adjusted HRs for all-cause and cardiopulmonary mortality according to years since cessation of solid fuel use for cooking by sex HRs were derived from Cox models for all-cause mortality (A–C), cardiovascular mortality (D–F), and respiratory mortality (G–I), stratified by sex (when appropriate) and study area and adjusted for age at baseline, education level, household income, smoking status, alcohol consumption, passive smoking, physical activity, body-mass index, diet (consumption of fresh fruit, preserved vegetables, and meat), cookstove ventilation, and solid fuel use for heating. Line at 1·0 represents the reference category persistent clean fuel users and previous solid fuel users with >10 years of cessation, indicating participants who had reported always using clean fuels or switching from solid to clean fuels for >10 years. Solid red lines show HR estimates and shaded areas show 95% CIs. HR=hazard ratio.

**Figure 2:**
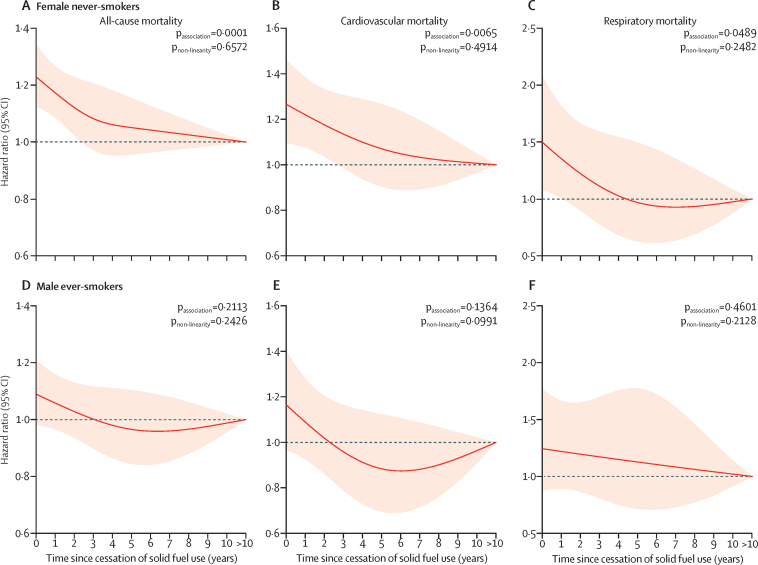
Adjusted HRs for all-cause and cardiopulmonary mortality according to years since cessation of solid fuel use for cooking among female never-smokers and male ever-smokers HRs were derived from Cox models for all-cause mortality (A, D), cardiovascular mortality (B, E), and respiratory mortality (C, F), stratified by study area and adjusted for age at baseline, education level, household income, alcohol consumption, passive smoking, physical activity, body-mass index, diet (consumption of fresh fruit, preserved vegetables, and meat), cookstove ventilation, and solid fuel use for heating. Line at 1·0 represents the reference category persistent clean fuel users and previous solid fuel users with >10 years of cessation, indicating participants who had reported always using clean fuels or switching from solid to clean fuels for >10 years. We only present results for female never-smokers (n=116 105; A–C) and male ever-smokers (n=41 506; D–F) because of the small sample size of female ever-smokers (n=5261) and male never-smokers (n=8805). Solid red lines show HR estimates and shaded areas show 95% CIs. HR=hazard ratio.

Risks of all-cause and cardiopulmonary mortality decreased swiftly after cessation of solid fuel use (figure 1), among both women and men. For persistent users of solid fuel, mortality risk was increased (for all-cause mortality, HR 1·15, 95% CI 1·08–1·22; for cardiovascular mortality, 1·19, 1·07–1·32; and for respiratory mortality, 1·41, 1·12–1·76) but fell at 5 years after cessation among previous users of solid fuel (respectively; 1·04, 0·96–1·12; 0·97, 0·85–1·10; and 1·10, 0·83–1·45), equivalent to more than a 60% reduction in excess risk. Risks of mortality continued to decrease with time afterwards. Similar patterns were seen in both women and men in stratified analyses, except no rapid decreasing trend in risk for respiratory mortality was noted among men in the first 5 years, possibly attributable to the small number of respiratory deaths among men, which restricted statistical power to generate reliable risk estimates (table 2; figure 1). When stratified by smoking status, associations remained significant when analyses were restricted to female never-smokers (figure 2); no statistically significant differences were noted in all-cause and cardiopulmonary mortality according to time since cessation of solid fuel use in male ever-smokers, probably because of a small sample size. Female ever-smokers and male never-smokers were not analysed because of small sample sizes.

**Figure 3:**
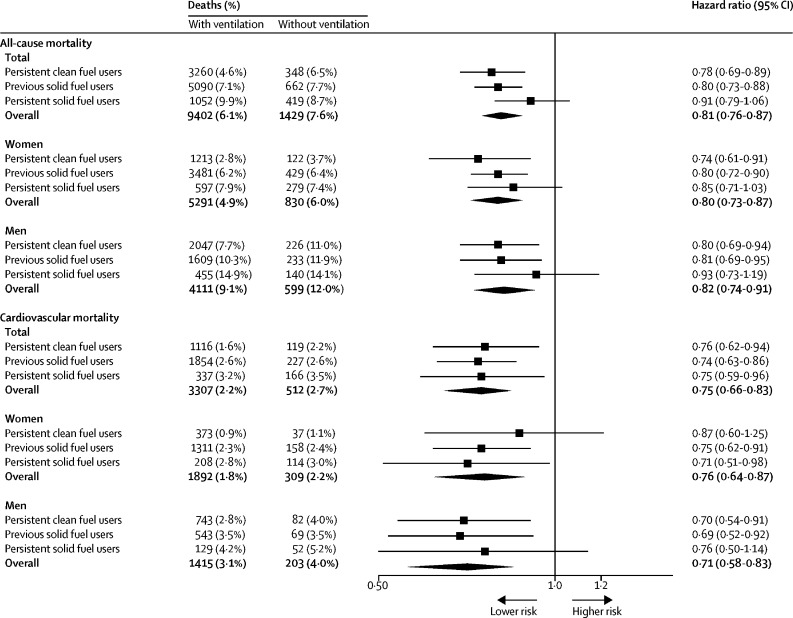
Adjusted HRs for all-cause and cardiovascular mortality according to use of ventilation by sex and types of cooking fuel HRs were stratified according to age at risk, sex (when appropriate), and study area and were adjusted for education level, household income, smoking status, alcohol consumption, passive smoking, physical activity, body-mass index, diet (consumption of fresh fruit, preserved vegetables, and meat), and solid fuel use for heating. Results for respiratory disease mortality are presented in the appendix (p 11) because of limited statistical power. HR=hazard ratio.

Participants cooking with ventilation had lower risks of all-cause mortality (HR 0·81, 95% CI 0·76–0·87) and cardiovascular mortality (0·75, 0·66–0·83) compared with those cooking without ventilation (figure 3), regardless of cooking fuel types. However, no such association was noted for respiratory mortality (appendix p 11). By examining specific disease subtypes, use of ventilation was associated with lower risks of death from ischaemic heart disease, total stroke, and haemorrhagic stroke (appendix p 12).

## Discussion

The findings of our prospective cohort study of people from five geographically diverse urban areas in China showed that use of solid fuel for cooking was associated with higher risk of all-cause and cardiopulmonary mortality compared with use of clean fuel. Cessation of solid fuel use was associated with lower mortality risk, with substantial risk reduction achieved within 5 years after cessation. Moreover, ventilation was associated with lower mortality risk, even among persistent users of clean fuel. To our knowledge, our study is the largest to date to investigate associations of cooking fuel types, time since switching to clean fuels, and use of ventilation with the risk for all-cause and cardiopulmonary mortality.

Findings of several prospective studies,^15^, ^16^, ^28^ including our previous study in rural China,^10^ have shown an increased mortality risk among users of solid fuel, which accord with our current study findings. Nevertheless, in the Prospective Urban and Rural Epidemiology (PURE) study of 91 350 participants (9·1 years of follow-up),^29^ when comparing solid fuel use versus clean fuel use in urban areas, no associations were seen for all-cause mortality (HR 1·12, 95% CI 0·96–1·31), cardiovascular mortality (1·06, 0·82–1·39), and respiratory mortality (1·26, 0·71–2·23), although the magnitude of risk estimates was roughly similar to those reported in our current study. With a sample size of urban participants approximately four times that of the PURE study (171 677 *vs* 43 001), we reported significantly higher risks for all-cause and cardiopulmonary mortality among persistent solid fuel users, and these findings were largely consistent across women and men.

Most previous studies have focused on mortality risks associated with current use of solid fuel. The association related to changes in mortality risk after cessation of solid fuel use remains largely uninvestigated. Two studies,^10^, ^15^ both including participants in the CKB Study, with one focusing on rural areas^10^ and the other on never-smokers,^15^ provided evidence that the mortality risk was lower after cessation of solid fuel use. These two studies, however, did not look at when mortality risk began to decrease after cessation. Only one study of 74 941 women from Shanghai^16^ investigated mortality risks in association with time since cessation of coal use (<10 years, 10–20 years, and >20 years), and reported that the risk of ischaemic heart disease mortality was reduced with increasing years of cessation, whereas no such trend was seen for all-cause mortality and death from other causes. By examining the association over a much finer timescale, our study has extended this previous research, and our findings showed that the excess risks of both all-cause and cardiopulmonary mortality among previous users of solid fuel decreased by more than 60% at 5 years after cessation, and continued to decrease afterwards. The discrepant findings from the Shanghai study might be because only about 1% of participants were persistent users of coal, and most had ceased use of coal at baseline. Moreover, inclusion of participants switching from coal use to non-cooking might bias the association, because those with pre-existing diseases were more likely to stop cooking but had higher mortality risks. Furthermore, as far as we know, we have shown for the first time a similar decreasing trend in mortality risk after switching to clean fuel use in both men and women. In summary, our findings underscore the urgency of transition from solid fuels to clean fuels for cooking, to minimise further adverse health effects. By focusing on urban areas, our findings have important public health implications globally because many low-income and middle-income countries are undergoing rapid economic development and experiencing an unprecedented pace of urbanisation, which provides an enormous opportunity to accelerate access to clean energy.^30^

Our study provides a unique finding that, even among users of clean fuels, use of ventilation was associated significantly with reduced risks of all-cause and cardiovascular mortality. In both our previous study in rural China^10^ and the PURE study,^29^ use of ventilation was associated with lower mortality risks among users of solid fuels. Our current study has, therefore, extended evidence for the health benefit of ventilation by showing that use of ventilation was associated with a lower risk of all-cause and cardiovascular mortality, even among those using clean fuels for cooking.

Our study has several limitations. First, classification of cooking fuel use might be inaccurate, because fuel use information was gathered by self-report; moreover, the three most recent residences might not cover early-life exposure. However, our resurvey of cooking fuel use showed reasonable reproducibility (weighted κ value of 0·56). Furthermore, we expect that not knowing about cooking fuel type during early life should not have a major effect on our results because the massive transition of cooking fuel types in China only started from the 1990s. Moreover, misclassification of fuel use would probably lead to underestimation rather than overestimation of the association between solid fuel use and mortality risk, because of the dilution effect of random measurement errors in the exposure.^31^ Second, although we have carefully adjusted for several covariates related to socioeconomic status (ie, educational level, household income, and occupation), residual confounding by unmeasured socioeconomic factors remains possible. Third, residual confounding might be present from concurrent exposure to ambient air pollution, to which household solid fuel use was a contributing source.^8^ Nevertheless, although we did not have data to adjust for ambient air quality, we stratified all analyses by study area, which was expected to at least partly account for ambient air pollution exposure, assuming a similar exposure level to participants from the same area. Finally, we only recorded the primary fuel used for cooking as the exposure, and we did not obtain data for secondary types of fuel use, types of cooking stoves, and effectiveness of ventilation facilities, all of which might affect the exposure level to solid fuel use. We expect to supplement this information in future resurveys and implement direct measurement of personal exposure to household air pollution to improve the exposure assessment.

In conclusion, our findings underscore the urgency of improving access to both clean fuels and ventilation facilities, which is especially promising for public health gains in low-income and middle-income countries, where an unprecedented pace of urbanisation is ongoing.

## Data sharing

Requests for data should be submitted to the China Kadoorie Biobank (CKB) Data Access Committee. As stated in the CKB data policy, the CKB Study Group (as data custodian) must maintain the integrity of the database for future use and regulate data access to comply with previous conditions agreed with the Chinese Government. Data security is an integral part of CKB Study protocols. Data can be released outside the CKB research group only with appropriate security safeguards.
