# Supplementary material for: Cooking fuels and risk of all-cause and cardiopulmonary mortality in urban China: a prospective cohort study
Source: Lancet Glob Health. 2020 Jan 20;8(3):e430–9. doi: 10.1016/S2214-109X(19)30525-X (PMC7031698; doi:10.1016/S2214-109X(19)30525-X)
Supplement: Supplementary appendix [file mmc1.pdf]

THE LANCET
Global Health

Supplementary appendix

This appendix formed part of the original submission and has been peer reviewed. We post it as supplied by the authors.

Supplement to: Yu K, Lv J, Qiu G, et al. Cooking fuels and risk of all-cause and cardiopulmonary mortality in urban China: a prospective cohort study. *Lancet Glob Health* 2020; published online Jan 19. http://dx.doi.org/10.1016/S2214-109X(19)30525-X.

Supplementary Appendix, Table of Contents

Page 2. List of study committees and investigators.
Page 3. Supplementary Methods.
Page 4. sTable 1: Reproducibility of Baseline Characteristics at Resurvey (N=8161).
Page 5. sTable 2: Adjusted Hazard Ratios for All-Cause and Cardiopulmonary Mortality According to Types of Cooking Fuels in the Sensitivity Analyses.
Page 6. sTable 3: Adjusted Hazard Ratios for All-Cause and Cardiopulmonary Mortality by Coal and Wood.
Page 7. sTable 4: Adjusted Hazard Ratios for Subtypes of Cardiopulmonary Mortality by Sex and Types of Cooking Fuels.
Page 8. sFigure 1: Locations of the Five Urban Study Areas of the China Kadoorie Biobank Study.
Page 9. sFigure 2: Flowchart of the Inclusion of Study Subjects.
Page 10. sFigure 3: Adjusted Hazard Ratios for All-cause Mortality in Association with Types of Cooking Fuels across Five Study Areas.
Page 11. sFigure 4: Adjusted Hazard Ratios of Ventilation Usage for Respiratory Mortality by Types of Cooking Fuels.
Page 12. sFigure 5: Adjusted Hazard Ratios of Ventilation Usage for Subtypes of Cardiopulmonary Mortality by Types of Cooking Fuels.

<u>Members of the China Kadoorie Biobank Collaborative Group</u>

International Steering Committee: Junshi Chen, Zhengming Chen (PI), Robert Clarke, Rory Collins, Yu Guo, Liming Li (PI), Jun Lv, Richard Peto, Robin Walters.

International Co-ordinating Centre, Oxford: Daniel Avery, Ruth Boxall, Derrick Bennett, Yumei Chang, Yiping Chen, Zhengming Chen, Robert Clarke, Huaidong Du, Simon Gilbert, Alex Hacker, Mike Hill, Michael Holmes, Andri Iona, Christiana Kartsonaki, Rene Kerosi, Ling Kong, Om Kurmi, Garry Lancaster, Sarah Lewington, Kuang Lin, John McDonnell, Iona Millwood, Qunhua Nie, Jayakrishnan Radhakrishnan, Paul Ryder, Sam Sansome, Dan Schmidt, Paul Sherliker, Rajani Sohoni, Becky Stevens, Iain Turnbull, Robin Walters, Jenny Wang, Lin Wang, Neil Wright, Ling Yang, Xiaoming Yang.

National Co-ordinating Centre, Beijing: Zheng Bian, Yu Guo, Xiao Han, Can Hou, Jun Lv, Pei Pei, Chao Liu, Yunlong Tan, Canqing Yu.

10 Regional Co-ordinating Centres: Qingdao CDC: Zengchang Pang, Ruqin Gao, Shanpeng Li, Shaojie Wang, Yongmei Liu, Ranran Du, Yajing Zang, Liang Cheng, Xiaocao Tian, Hua Zhang, Yaoming Zhai, Feng Ning, Xiaohui Sun, Feifei Li. **Licang CDC:** Silu Lv, Junzheng Wang, Wei Hou. **Heilongjiang Provincial CDC:** Mingyuan Zeng, Ge Jiang, Xue Zhou. **Nangang CDC:** Liqiu Yang, Hui He, Bo Yu, Yanjie Li, Qinai Xu, Quan Kang, Ziyan Guo. **Hainan Provincial CDC:** Dan Wang, Ximin Hu, Hongmei Wang, Jinyan Chen, Yan Fu, Zhenwang Fu, Xiaohuan Wang. **Meilan CDC:** Min Weng, Zhendong Guo, Shukuan Wu, Yilei Li, Huimei Li, Zhifang Fu. **Jiangsu Provincial CDC:** Ming Wu, Yonglin Zhou, Jinyi Zhou, Ran Tao, Jie Yang, Jian Su. **Suzhou CDC:** Fang liu, Jun Zhang, Yihe Hu, Yan Lu, Liangcai Ma, Aiyu Tang, Shuo Zhang, Jianrong Jin, Jingchao Liu. **Guangxi Provincial CDC:** Zhenzhu Tang, Naying Chen, Ying Huang. **Liuzhou CDC:** Mingqiang Li, Jinhuai Meng, Rong Pan, Qilian Jiang, Jian Lan, Yun Liu, Liuping Wei, Liyuan Zhou, Ningyu Chen Ping Wang, Fanwen Meng, Yulu Qin, Sisi Wang. **Sichuan Provincial CDC:** Xianping Wu, Ningmei Zhang, Xiaofang Chen, Weiwei Zhou. **Pengzhou CDC:** Guojin Luo, Jianguo Li, Xiaofang Chen, Xunfu Zhong, Jiaqiu Liu, Qiang Sun. **Gansu Provincial CDC:** Pengfei Ge, Xiaolan Ren, Caixia Dong. **Maiji CDC:** Hui Zhang, Enke Mao, Xiaoping Wang, Tao Wang, Xi zhang. **Henan Provincial CDC:** Ding Zhang, Gang Zhou, Shixian Feng, Liang Chang, Lei Fan. **Huixian CDC:** Yulian Gao, Tianyou He, Huarong Sun, Pan He, Chen Hu, Xukui Zhang, Huifang Wu, Pan He. **Zhejiang Provincial CDC:** Min Yu, Ruying Hu, Hao Wang. **Tongxiang CDC:** Yijian Qian, Chunmei Wang, Kaixu Xie, Lingli Chen, Yidan Zhang, Dongxia Pan, Qijun Gu. **Hunan Provincial CDC:** Yuelong Huang, Biyun Chen, Li Yin, Huilin Liu, Zhongxi Fu, Qiaohua Xu. **Liuyang CDC:** Xin Xu, Hao Zhang, Huajun Long, Xianzhi Li, Libo Zhang, Zhe Qiu.

Supplementary Methods
Statistical analysis

Among the 226,186 urban participants, we further excluded 179 participants whose three recent residential periods summed up to be greater than their age, 54,317 participants who did not cooking regulary (monthly [n=9126], or rarely/never [n=45,191]), and 74 participants who used other unspecified fuels for cooking, leaving 171,677 participants in the final analyses (Fig. S2 in the Appendix). Baseline characteristics of the study population are presented as means with standard deviations (SDs) or percentages by types of cooking fuels. The reproducibility of baseline information, including cooking fuel use, were evaluated through intraclass correlation coefficients (ICC) for continuous variables and the weighted κ statistic for categorical variables using repeated measures at baseline and in the resurvey. Cox proportional hazards regression models were used to estimate multivariate-adjusted hazard ratios (HRs) and 95% confidence intervals (CIs) of mortality risks associated with types of cooking fuels, time since cessation of solid fuel use, and the use of ventilation. All models were stratified by age-at-risk (in 5-year intervals, 12 strata), sex (men/women, 2 strata), and study areas (5 strata), and were adjusted for established risk factors including education (primary school or below, middle school, and high school or above), household income (<20,000, 20,000-34,999, and ≥35,000 yuan/year), smoking status (current, former, and never smoker), passive smoking (yes/no), drinking (ever/never), body mass index (BMI, calculated as weight in kilograms divided by height in meters squared), physical activity (metabolic equivalent of tasks hours/day), dietary factors (daily consumption of fresh fruit, preserved vegetables, and meat), as well as cookstove ventilation (with/without) and types of heating fuel use (not heating, clean fuels, solid fuels, and other unspecified fuels). We tested the proportional hazard assumption using the Schoenfeld residuals and found no evidence of departure from the assumption in all presented models (P=0·0787, 0·0810, and 0·2595 for all-cause, cardiovascular, and respiratory mortality, respectively).

We then constructed spline curves using the restricted cubic spline function in a stratified Cox model with the %LGTPHCURV9 SAS macro to visually inspect the change of mortality risk along with time since cessation of solid fuel use with mortality risk, where the exposure variable was the time since cessation of solid fuel use, with persistent solid fuel users assigned a value of 0 in time since cessation of solid fuel use. The curve was first plotted against the entire time range (1-60 years) to visually inspect when mortality risk began to decrease to the level of persistent clean fuel users, which was at the 8th to 10th year. Persistent clean fuel users were then assigned a value of 11 and served as the reference group together with those who had ceased use for at least 11 years. We set three knots at 5, 50, and 95 percentiles of the exposure variable for the restricted cubic spline function, with no offset values for the fixed terms, resulting in two degrees of freedom. We included sex and study areas as stratifying variables, and age, education, household income, smoking status, passive smoking, drinking, BMI, physical activity, dietary factors, cookstove ventilation and solid fuel use for heating as covariates. Non-linearity was examined by a likelihood ratio test, which compared two models with and without nonlinear terms. We also performed separate analyses among men and women, and across subgroups of smoking status considering the possible joint effect of smoking and solid fuel use on mortality risk. In all analyses, exposure variables were assessed using fuel use information reported at baseline, without considering fuel use changes occurring during follow-up.

To test the robustness of our HR estimates of mortality risks associated with types of cooking fuels, we conducted several sensitivity analyses, including: 1) further adjusting for conventional cardiovascular risk factors including hypertension and diabetes which could be potential confounders or mediators, or further adjusting for other variables including occupation, self-rated health status, family history of cardiovascular disease, and survey season; 2) excluding those with major diseases (including cardiovascular disease and cancer) at baseline; 3) excluding participants who died within the first two years of follow-up to scrutinize reverse causality, because there was a possibility that these participants had subclinical diseases at baseline, which could affect their choice of cooking fuel types; 4) examining coal use and wood use separately; and 5) examining specific causes of death, including ischaemic heart disease (IHD), stroke and its ischaemic and haemorrhagic subtypes, chronic obstructive pulmonary disease (COPD), and pneumonia. All sensitivity analyses were conducted using Cox regression with the same stratification and covariates as in the main model.

Two-sided P values <0·05 were considered statistically significant. All analyses were performed using SAS version 9.4 (SAS Institute Inc), and graphs were plotted using R version 3.4.2 (R Foundation).

Supplementary Table 1: Reproducibility of Baseline Characteristics at Resurvey (N=8161). *

Characteristics	Baseline	Resurvey	ICC (95% CI)	Weighted κ (95% CI)
**Education, %**				0·89 (0·88-0·90)
Primary school or below	2802 (34·3)	2816 (34·5)		
Middle school	2512 (30·8)	2476 (30·3)		
High school or above	2847 (34·9)	2869 (35·2)		
**Household income, %**				0·43 (0·42-0·45)
<20,000 yuan/year	3781 (46·3)	2456 (30·1)		
20,000-34,999 yuan/year	2535 (31·1)	2483 (30·4)		
⩾35,000 yuan/year	1845 (22·6)	3222 (39·5)		
**Smoking, %**				0·88 (0·87-0·89)
Never	5448 (66·8)	5587 (68·5)		
Previous	539 (6·6)	566 (6·9)		
Current	2174 (26·6)	2008 (24·6)		
**Drinking, %**				0·61 (0·59-0·63)
Never	3646 (44·7)	3802 (46·6)		
Previous	114 (1·4)	120 (1·5)		
Current	4401 (53·9)	4239 (51·9)		
**Cooking fuel use, %** †				0·56 (0·54-0·58)
Not cooking	1916 (23·5)	2038 (25·0)		
Clean fuels	5631 (69·0)	5700 (69·8)		
Solid fuels	611 (7·5)	420 (5·1)		
Unspecific fuels	3 (0·0)	3 (0·0)		
**Heating fuel use, %**				0·81 (0·79-0·83)
Not heating	3806 (46·6)	3912 (47·9)		
Clean fuels	3301 (40·4)	3380 (41·4)		
Solid fuels	1044 (12·8)	867 (10·6)		
Unspecific fuels	10 (0·1)	2 (0·0)		
**Ventilation status, %** ‡				0·55 (0·53-0·57)
Yes	6023 (73·8)	6654 (81·6)		
Not all stoves	1162 (14·3)	435 (5·4)		
No	958 (11·8)	1054 (12·9)		
**Physical activity, means ± SD, MET-hour/d ***	17·9 ± 12·5	16·7 ± 12·2	0·74 (0·72-0·75)	
**Body mass index, means ± SD, kg/m^2**	24·4 ± 3·5	24·4 ± 3·4	0·969 (0·968-0·971)	

* MET-h/d = Metabolic Equivalent of Tasks - hours per day. Weighted κ value was calculated for categorical variables and intraclass correlation coefficient (ICC) was calculated for continuous variables.
† 6431 (78·8%) of the participants in the resurvey reported the same cooking fuel use as at baseline.
‡ 18 partipants who reported no cooking facility lacked information of ventilation status. 6594 (80·8%) of the participants in the resurvey reported the same ventilation use as at baseline.

Supplementary Table 2: Adjusted Hazard Ratios for All-Cause and Cardiopulmonary Mortality According to Types of Cooking Fuels in the Sensitivity Analyses.

Causes of death	No. of Death (%)	Main model*	With extended adjustment for conventional CVD risk factors†	With extended adjustment for other variables‡	Excluding participants with major diseases at baseline§	Excluding deaths occurred within the first two years of follow-up
**All-cause**						
Persistent clean fuel user	3608 (4·8)	Ref.	Ref.	Ref.	Ref.	Ref.
Previous solid fuel user	5752 (7·1)	0·97 (0·93-1·01)	0·97 (0·93-1·01)	0·96 (0·92-1·01)	0·96 (0·91-1·01)	0·97 (0·93-1·01)
Persistent solid fuel user	1471 (9·6)	1·19 (1·10-1·28)	1·19 (1·11-1·28)	1·13 (1·05-1·21)	1·14 (1·06-1·36)	1·16 (1·08-1·24)
**Cardiovascular disease**						
Persistent clean fuel user	1235 (1·6)	Ref.	Ref.	Ref.	Ref.	Ref.
Previous solid fuel user	2081 (2·6)	0·98 (0·91-1·05)	0·98 (0·91-1·06)	0·97 (0·90-1·04)	0·97 (0·89-1·06)	0·98 (0·91-1·05)
Persistent solid fuel user	503 (3·3)	1·24 (1·10-1·39)	1·26 (1·11-1·42)	1·21(1·07-1·36)	1·20 (1·06-1·36)	1·24 (1·10-1·39)
**Respiratory disease**						
Persistent clean fuel user	209 (0·3)	Ref.	Ref.	Ref.	Ref.	Ref.
Previous solid fuel user	415 (0·5)	1·08 (0·90-1·29)	1·08 (0·90-1·29)	1·07 (0·89-1·26)	1·09 (0·89-1·33)	1·08 (0·90-1·29)
Persistent solid fuel user	137 (0·9)	1·43 (1·10-1·85)	1·52 (1·20-1·94)	1·42 (1·11-1·82)	1·45 (1·11-1·89)	1·49 (1·17-1·91)

*Cox regression models were stratified by age-at-risk, sex, and study area, and were adjusted for education level, household income, alcohol consumption, smoking status, passive smoking, physical activity, BMI, cookstove ventilation and solid fuel use for heating. †Additionally adjusted for hypertension and type 2 diabetes. ‡Additionally adjusted for occupation, self-rated health, family history of cardiovascular disease, and survey season. §Major diseases included CVD and cancer.

Supplementary Table 3: Adjusted Hazard Ratios for All-Cause and Cardiopulmonary Mortality by Coal and Wood.

Causes of death	N	All-cause mortality		Cardiovascular mortality		Respiratory mortality	
		No. of Deaths	Hazard Ratio (95% CI)	No. of Deaths	Hazard Ratio (95% CI)	No. of Deaths	Hazard Ratio (95% CI)
**Total**							
**Persistent clean fuel user**	75785	3608	Ref.	1235	Ref.	209	Ref.
**Persistent coal user**	2766	304	1·11 (0·98-1·24)	126	1·19 (1·00-1·42)	34	1·49 (1·00-2·23)
**Persistent wood user**	12615	1167	1·24 (1·16-1·33)	377	1·32 (1·17-1·49)	103	1·36 (1·07-1·73)
**Women**							
**Persistent clean fuel user**	47077	1335	Ref.	410	Ref.	66	Ref.
**Persistent coal user**	2040	199	1·16 (1·00-1·34)	80	1·16 (0·93-1·46)	24	1·81 (1·18-2·77)
**Persistent wood user**	9292	677	1·29 (1·17-1·42)	242	1·39 (1·18-1·63)	53	1·37 (0·96-1·95)
**Men**							
**Persistent clean fuel user**	28708	2273	Ref.	825	Ref.	143	Ref.
**Persistent coal user**	726	105	1·03 (0·84-1·25)	46	1·23 (0·91-1·65)	10	1·07 (0·57-2·01)
**Persistent wood user**	3323	490	1·20 (1·08-1·33)	135	1·24 (1·03-1·50)	50	1·35 (0·97-1·88)

Hazard ratios were derived from Cox models stratified by age-at-risk, sex (where appropriate) and study area, and adjusted for education level, household income, alcohol consumption, smoking status, passive smoking, physical activity, BMI, diet (consumption of fresh fruit, preserved vegetables, and meat), cookstove ventilation and types of heating fuel use.

Supplementary Table 4: Adjusted Hazard Ratios for Subtypes of Cardiopulmonary Mortality by Sex and Types of Cooking Fuels.

Causes of death	N	Ischaemic heart disease		Stroke		Ischaemic stroke		Haemorrhagic stroke		COPD		Pneumonia	
		No. of Deaths	Hazard Ratio (95% CI)	No. of Deaths	Hazard Ratio (95% CI)	No. of Deaths	Hazard Ratio (95% CI)	No. of Deaths	Hazard Ratio (95% CI)	No. of Deaths	Hazard Ratio (95% CI)	No. of Deaths	Hazard Ratio (95% CI)
**Total**													
**Persistent clean fuel user**	75785	610	Ref.	376	Ref.	146	Ref.	195	Ref.	76	Ref.	87	Ref.
**Previous solid fuel user**	80511	940	0·95 (0·86-1·07)	652	0·98 (0·85-1·12)	251	0·94 (0·75-1·16)	319	1·04 (0·86-1·26)	177	1·22 (0·92-1·62)	169	0·99 (0·75-1·30)
<5 years since cessation	7728	60	1·00 (0·89-1·12)	73	1·07 (0·84-1·36)	28	1·27 (0·87-1·86)	32	1·15 (0·85-1·56)	23	1·39 (0·89-2·17)	9	0·93 (0·53-1·63)
5-10 years since cessation	17927	153	0·90 (0·74-1·09)	144	1·14 (0·93-1·41)	51	1·01 (0·71-1·45)	67	1·01 (0·70-1·44)	42	1·34 (0·88-2·05)	23	0·65 (0·38-1·11)
>10 years since cessation	54856	727	0·81 (0·63-1·04)	435	0·97 (0·83-1·12)	172	0·95 (0·75-1·20)	220	1·04 (0·85-1·29)	112	1·15 (0·84-1·57)	137	1·07 (0·80-1·43)
**Persistent solid fuel user**	15381	127	1·03 (0·84-1·27)	214	1·38 (1·15-1·67)	72	1·39 (1·02-1·90)	109	1·59 (1·22-2·07)	87	1·79 (1·27-2·52)	25	1·01 (0·62-1·64)
**Women**													
**Persistent clean fuel user**	47077	208	Ref.	131	Ref.	47	Ref.	66	Ref.	24	Ref.	26	Ref.
**Previous solid fuel user**	62957	682	1·00 (0·85-1·18)	445	1·08 (0·88-1·33)	175	1·05 (0·75-1·46)	210	1·20 (0·89-1·61)	101	1·02 (0·64-1·63)	111	1·07 (0·69-1·67)
<5 years since cessation	5867	48	1·01 (0·85-1·19)	42	1·10 (0·78-1·54)	16	1·42 (0·84-2·38)	20	1·47 (0·98-2·21)	13	1·18 (0·61-2·28)	5	0·98 (0·45-2·13)
5-10 years since cessation	13803	102	0·87 (0·68-1·13)	102	1·38 (1·04-1·83)	38	1·17 (0·73-1·88)	44	1·09 (0·65-1·81)	20	0·91 (0·47-1·75)	14	0·65 (0·31-1·37)
>10 years since cessation	43287	532	0·93 (0·69-1·26)	301	1·05 (0·85-1·30)	121	1·02 (0·71-1·44)	146	1·17 (0·86-1·59)	68	0·95 (0·59-1·54)	92	1·17 (0·75-1·85)
**Persistent solid fuel user**	11332	87	1·09 (0·82-1·43)	127	1·52 (1·15-2·01)	43	1·43 (0·90-2·26)	66	2·05 (1·38-3·03)	50	1·83 (1·06-3·16)	18	1·37 (0·70-2·68)
**Men**													
**Persistent clean fuel user**	28708	402	Ref.	245	Ref.	99	Ref.	129	Ref.	52	Ref.	61	Ref.
**Previous solid fuel user**	17554	258	0·91 (0·78-1·07)	207	0·94 (0·78-1·13)	76	0·90 (0·66-1·22)	109	0·98 (0·75-1·27)	76	1·46 (1·02-2·09)	58	0·95 (0·66-1·37)
<5 years since cessation	1861	12	0·95 (0·80-1·14)	31	1·11 (0·78-1·59)	12	1·14 (0·63-2·07)	12	1·00 (0·59-1·70)	10	1·54 (0·83-2·86)	4	0·93 (0·40-2·17)
5-10 years since cessation	4124	51	0·94 (0·69-1·29)	42	0·86 (0·60-1·23)	13	0·80 (0·44-1·47)	23	0·85 (0·51-1·40)	22	1·91 (1·10-3·32)	9	0·71 (0·32-1·57)
>10 years since cessation	11569	195	0·57 (0·36-0·92)	134	0·90 (0·72-1·12)	51	0·87 (0·61-1·23)	74	0·97 (0·72-1·31)	44	1·27 (0·84-1·93)	45	1·01 (0·67-1·50)
**Persistent solid fuel user**	4049	40	0·95 (0·67-1·33)	87	1·35 (1·04-1·76)	29	1·45 (0·93-2·26)	43	1·37 (0·94-1·99)	37	1·67 (1·06-2·65)	7	0·69 (0·31-1·55)

Hazard ratios were derived from Cox models stratified by age-at-risk, sex (where appropriate) and study area, and adjusted for education level, household income, alcohol consumption, smoking status, passive smoking, physical activity, BMI, diet (consumption of fresh fruit, preserved vegetables, and meat), cookstove ventilation and types of heating fuel use.

Supplementary Figure 1: Locations of the Five Urban Study Areas of the China Kadoorie Biobank Study.

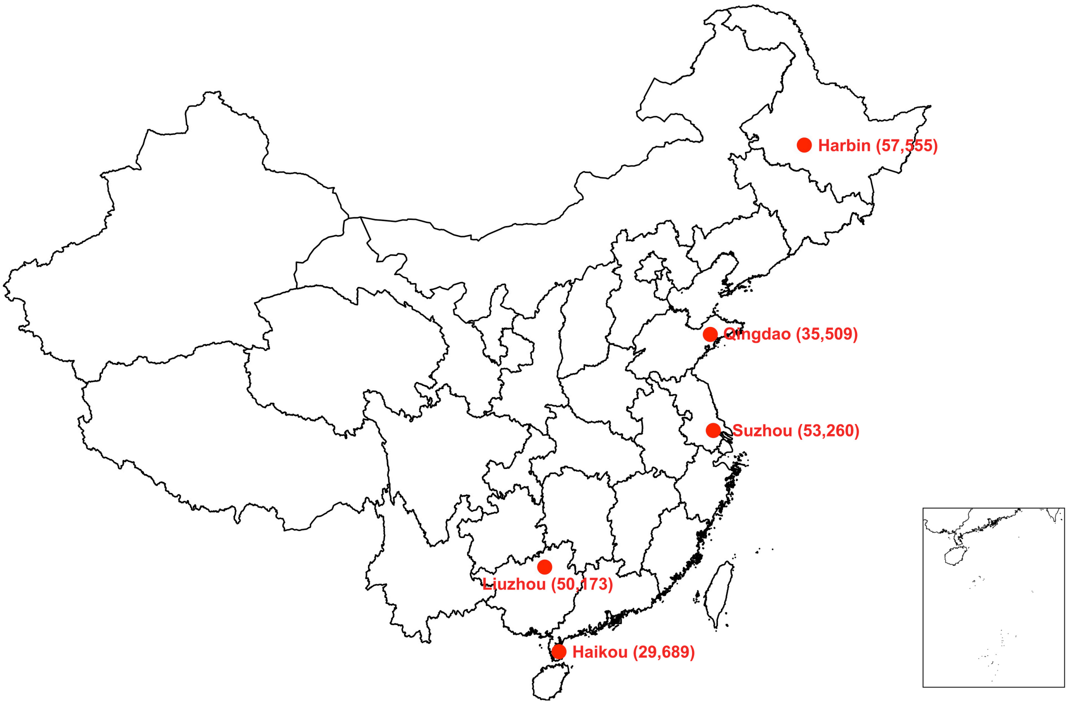

Solid circles are the urban areas included in the present study of the China Kadoorie Biobank. Number recruited at baseline in each site is shown in brackets.

Supplementary Figure 2: Flowchart of the Inclusion of Study Subjects.

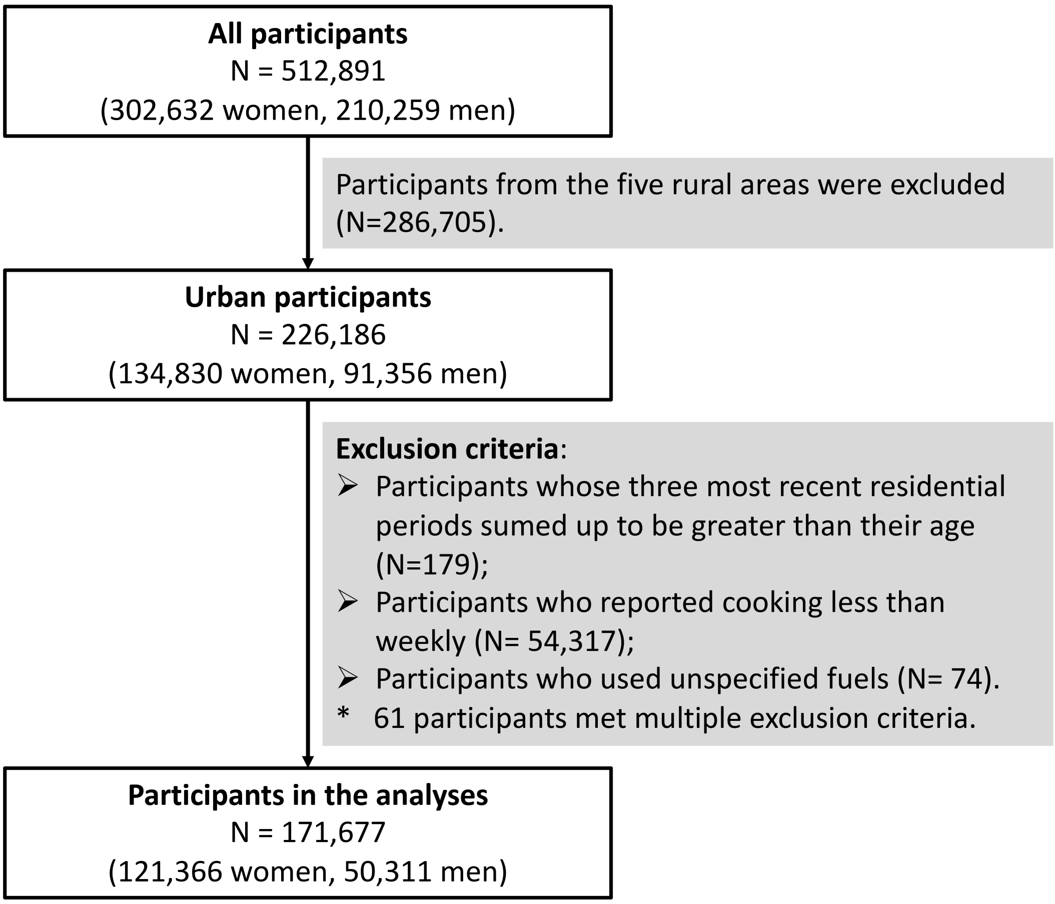

Supplementary Figure 3: Adjusted Hazard Ratios for All-cause and Cardiovascular Mortality in Association with Types of Cooking Fuels across Five Study Areas.

Study areas	A. All-cause mortality: No. of Deaths (%)	A. All-cause mortality: Hazard ratio (95% CI)	B. Cardiovascular mortality: No. of Deaths (%)	B. Cardiovascular mortality: Hazard ratio (95% CI)
**Previous solid fuel users**				
Harbin	2007 (9·9)	1·12 (0·95-1·31)	801 (3·9)	0·95 (0·84-1·07)
Qingdao	518 (7·2)	1·02 (0·95-1·10)	204 (2·8)	1·07 (0·88-1·30)
Suzhou	1089 (4·5)	0·92 (0·81-1·04)	259 (1·1)	0·90 (0·69-1·18)
Liuzhou	1899 (8·2)	0·94 (0·86-1·02)	714 (3·1)	0·95 (0·83-1·10)
Haikou	239 (4·4)	1·05 (0·88-1·25)	103 (1·9)	1·21 (0·92-1·60)
**Overall**	**5752 (7·1)**	**0·98 (0·94-1·03)**	**2081 (2·6)**	**0·97 (0·90-1·05)**
P for heterogeneity		0·189		0·503
**Persistent solid fuel users**				
Harbin	99 (11·9)	1·05 (0·85-1·29)	42 (5·1)	1·10 (0·89-1·35)
Qingdao	13 (7·7)	0·65 (0·38-1·14)	5 (3·0)	0·69 (0·28-1·67)
Suzhou	798 (10·1)	1·17 (0·97-1·40)	244 (3·1)	1·41 (1·11-1·78)
Liuzhou	235 (13·5)	1·10 (1·00-1·21)	87 (5·0)	1·03 (0·80-1·33)
Haikou	326 (6·9)	1·12 (0·95-1·33)	125 (2·6)	1·26 (0·98-1·63)
**Overall**	**1471 (9·6)**	**1·09 (1·01-1·17)**	**503 (3·3)**	**1·14 (1·01-1·28)**
P for heterogeneity		0·208		0·256

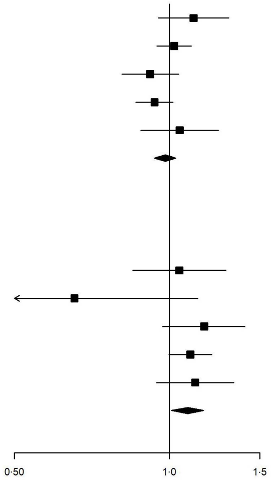

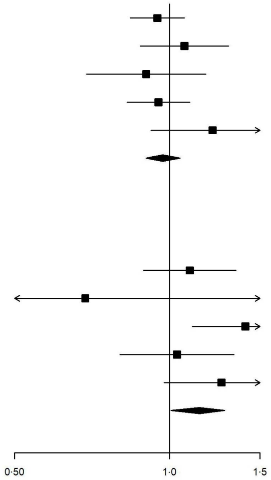

Hazard ratios were stratified according to age-at-risk and sex, and were adjusted for education level, household income, alcohol consumption, smoking status, passive smoking, physical activity, BMI, diet (consumption of fresh fruit, preserved vegetables, and meat), cookstove ventilation and solid fuel use for heating

Supplementary Figure 4: Adjusted Hazard Ratios of Ventilation Usage for Respiratory Mortality by Types of Cooking Fuels.

	No. of Deaths (%)			Hazard ratio (95% CI)
**Total**	**With ventilation**	**Without ventilation**		
Persistent clean fuel user	182 (0·3)	27 (0·5)		0·66 (0·41-1·06)
Previous solid fuel user	381 (0·5)	34 (0·4)		1·10 (0·74-1·63)
Persistent solid fuel user	96 (0·9)	41 (0·9)		1·09 (0·68-1·75)
**Overall**	**659 (0·4)**	**102 (0·5)**		**1·02 (0·80-1·29)**

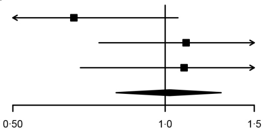

Hazard ratios were derived from Cox models stratified by age-at-risk, sex and study area, and adjusted for education level, household income, smoking status, alcohol consumption, passive smoking, physical activity, BMI, diet (consumption of fresh fruit, preserved vegetables, and meat), and solid fuel use for heating.

Supplementary Figure 5: Adjusted Hazard Ratios of Ventilation Usage for Subtypes of Cardiopulmonary Mortality Types of Cooking Fuels.

	No. of Deaths (%) With ventilation	No. of Deaths (%) Without ventilation	Hazard ratio (95% CI)
**A. Ischaemic heart disease**			
Persistent clean fuel user	564 (0·8)	46 (0·9)	0·72 (0·52-1·00)
Previous solid fuel user	874 (1·2)	66 (0·8)	0·75 (0·57-0·99)
Persistent solid fuel user	68 (0·6)	59 (1·2)	0·69 (0·44-1·08)
**Overall**	**1506 (1·0)**	**171 (0·9)**	**0·74 (0·62-0·89)**
**B. Stroke**			
Persistent clean fuel user	336 (0·5)	40 (0·7)	0·71 (0·55-1·07)
Previous solid fuel user	564 (0·8)	88 (1·0)	0·67 (0·45-0·98)
Persistent solid fuel user	145 (1·4)	69 (1·4)	0·93 (0·73-1·20)
**Overall**	**1045 (0·7)**	**197 (1·0)**	**0·78 (0·65-0·93)**
**C. Ischaemic stroke**			
Persistent clean fuel user	132 (0·2)	14 (0·3)	0·66 (0·36-1·23)
Previous solid fuel user	228 (0·1)	23 (0·3)	0·98 (0·61-1·60)
Persistent solid fuel user	47 (0·4)	25 (0·5)	0·97 (0·51-1·85)
**Overall**	**407 (0·3)**	**62 (0·3)**	**0·91 (0·66-1·24)**
**D. Haemorrhagic stroke**			
Persistent clean fuel user	172 (0·2)	23 (0·4)	0·71 (0·43-1·16)
Previous solid fuel user	280 (0·4)	39 (0·5)	0·66 (0·45-0·97)
Persistent solid fuel user	68 (0·6)	41 (0·9)	0·46 (0·28-0·77)
**Overall**	**520 (0·3)**	**103 (0·5)**	**0·71 (0·56-0·91)**
**E. COPD**			
Persistent clean fuel user	59 (0·1)	17 (0·3)	0·50 (0·26-0·97)
Previous solid fuel user	154 (0·2)	23 (0·3)	1·04 (0·62-1·72)
Persistent solid fuel user	63 (0·6)	24 (0·5)	1·24 (0·65-2·37)
**Overall**	**276 (0·2)**	**64 (0·3)**	**0·97 (0·71-1·33)**
**F. Pneumonia**			
Persistent clean fuel user	83 (0·1)	4 (0·1)	1·52 (0·52-4·46)
Previous solid fuel user	162 (0·2)	7 (0·1)	1·28 (0·58-2·86)
Persistent solid fuel user	16 (0·2)	9 (0·2)	0·99 (0·36-2·78)
**Overall**	**261 (0·2)**	**20 (0·1)**	**1·30 (0·78-2·18)**

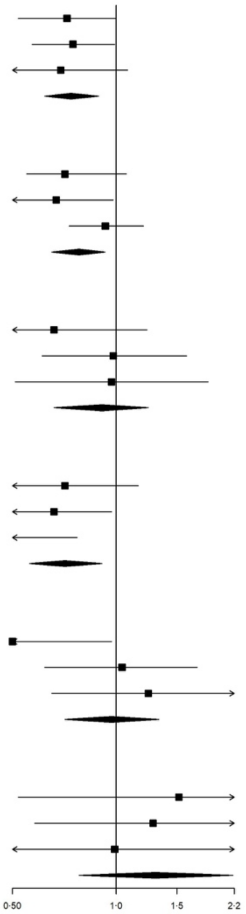

Hazard ratios were derived from Cox models stratified by age-at-risk, sex and study area, and adjusted for education level, household income, alcohol consumption, smoking status, passive smoking, physical activity, BMI, diet (consumption of fresh fruit, preserved vegetables, and meat), and types of heating fuel use.
